# Cerebellar ataxia and exercise intolerance in Erdheim-Chester disease

**DOI:** 10.1186/s40673-020-00125-x

**Published:** 2021-01-06

**Authors:** Eleonora Lauricella, Antonio d’Amati, Giuseppe Ingravallo, Maurizio Foresio, Domenico Ribatti, Marina de Tommaso, Mauro Cives, Francesco Girolamo

**Affiliations:** 1 Department of Biomedical Sciences and Human Oncology, Clinical Oncology Section, University of Bari School of Medicine, Bari, Italy; 2 Department of Emergency and Organ Transplantation (D.E.T.O.), Pathology Section, University of Bari School of Medicine, Bari, Italy; 3 U.O.C. Urologia, Ospedale SS Annunziata, Taranto, Italy; 4 Department of Basic Medical Sciences, Neurosciences and Sense Organs, Human Anatomy Section, University of Bari School of Medicine, Policlinico Universitario, Piazza Giulio Cesare, 11, 70124 Bari, Italy; 5 National Cancer Research Center, Tumori Institute “Giovanni Paolo II”, Bari, Italy

**Keywords:** Ataxia, Cerebellar atrophy Cognitive impairment, Erdheim-Chester disease, Fatigability, Histiocytosis

## Abstract

**Background:**

Erdheim-Chester disease (ECD), a rare disorder of monocyte/macrophage lineage, has been related to cerebellar dysfunction. To increase the awareness of this rare, protean disease, an unusual, myasthenia-like onset of ECD is reported.

**Case presentation:**

A 42-year-old man presented with a 6-year history of mild evening fatigability in his four limbs followed by motor and cognitive symptoms associated with cerebellar atrophy, dentate nuclei and dentato-thalamic pathway degeneration. Magnetic resonance imaging revealed hyperintense signals in T2 and fluid-attenuated inversion recovery sequences within the pons, cerebellar white matter, dentate nuclei and globi pallidi in the absence of any contrast enhancement. Whole-body bone scintigraphy with ^99^Technetium - methylene diphosphonate and fluorodeoxyglucose-positron emission tomography both revealed symmetric uptake in the lower extremities a finding suggestive of a diagnosis of ECD. Histological examination revealed diffuse infiltration of CD 68^+^ histiocytes with foamy cytoplasms in the presence of B-type of Rapidly Accelerated Fibrosarcoma protein kinase *(BRAF)*^V600E^ activating mutation in tumor cells.

**Conclusion:**

In patients with myasthenia-like symptoms who test negatively for myasthenia gravis, neurodegenerative diseases, and disorders of the hypothalamus, a diagnosis of ECD should be taken into consideration.

## Background

Erdheim-Chester disease (ECD) is a histiocytosis characterized by the formation of xanthogranulomatous lesions in the long bones, cardiovascular system, retroperitoneum, skin, lung, and central nervous system (CNS) [1–4]. It is an exceedingly rare clonal hematopoietic disorder of monocyte/macrophages and dendritic cell lineages, primarily affecting male subjects in midlife [3, 4]. Mutations activating the mitogen-activated protein kinase pathway are found in more than 80% of patients with ECD, being Rapidly Accelerated Fibrosarcoma protein kinase (*BRAF*)^*V600E*^ activating mutations in 57–70% of cases, and mitogen-activated protein kinase kinase 1 (*MAP2K1*) mutations in approximately 20% of cases [5]. One third of ECD patients initially presents with neurological symptoms, especially diabetes insipidus, ataxia, dysarthria, cognitive or visual impairment [3, 6, 7], and exclusive CNS involvement can last for months [5]. The diagnosis of ECD is often challenging, due to its rare incidence and protean manifestations with sequential multi-organ involvement. Neurologic involvement is independently predictive of poor prognosis in ECD patients [1], and timely treatment of neuro-ECD can improve outcomes [8]. Here, we report a single case of ECD with an unusual neurological presentation, in order to increase the awareness of this rare disease, as well as to describe aspects supporting the clinical evaluation and management of ECD patients.

## Case presentation

A 42-year-old man presented with a 6-year history of mild evening fatigability in all four limbs followed by slowly progressive gait imbalance and recurrent diplopia, despite an unremarkable cerebral and orbital magnetic resonance imaging (MRI) as well as negative anti-acetylcholine receptor antibody serology, repetitive nerve-stimulation and single fiber electromyography. During the last 3 years, he had progressively developed limb incoordination, slurred speech and dysphagia, and lost approximately 20% of his body weight. His family history was positive for rheumatoid arthritis, Sjögren’s syndrome, pancreatic cancer, but negative for ataxia, myasthenia gravis, neuropathy, tremors, and dementia. His medical history was significant for allergic asthma, increased blood pressure, and left retinal macular degeneration. At the time of presentation to our Institution, the general physical examination was unremarkable apart from bilateral xanthelasma, vitiligo on the dorsal aspect of hand fingers and yellowish papulae on both feet and the left pectoral region. Neurologic examination revealed acalculia (mini mental state examination: 23/30), horizontal gaze nystagmus, normal fundoscopic and pupillary examinations, slow start, hypermetric saccades mainly in the horizontal plane and mild bilateral neurosensorial hypoacusia. Romberg’s sign was present, and a marked ataxic gait, tight-rope walking inability, dysdiadochokinesia, arms dysmetria, tremor, dysarthria, rhinolalia, and motor hindrance of the hands were also observed.

**Fig. 1:**
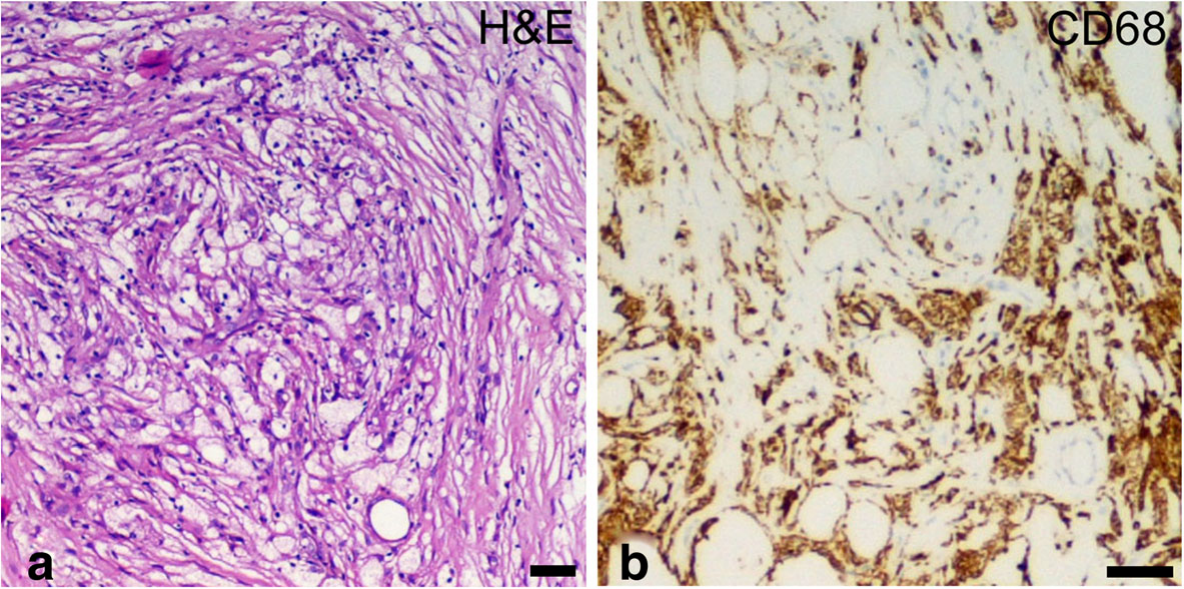
Histopathologic findings suggestive of Erdheim-Chester disease (ECD). Histological examination of skin papulae showing foamy macrophages (**a**; hematoxylin and eosin: H&E) positive for CD68 (**b**). Scale bars: 10 μm

A biopsy of one of the skin papulae showed nodular infiltration of the dermis by mononuclear histiocytes (CD163^+^, CD68^+^, CD14^+^, CD4^+^, CD1a^−^, CD207^−^, S100^−^, phosphorylated extracellular signal-regulated kinases^+^). Several histiocytes showed a foamy cytoplasm (Fig. 1).

**Fig. 2:**
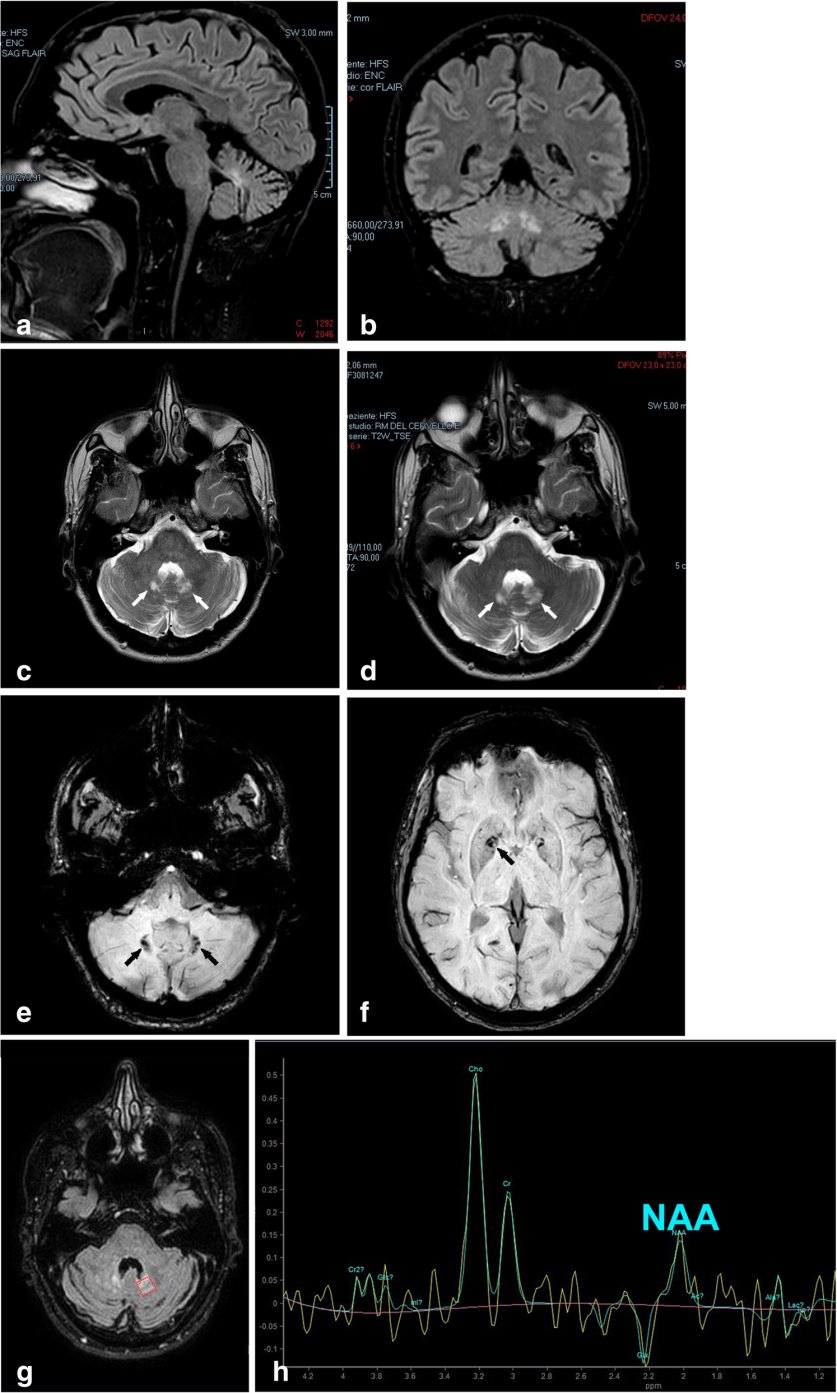
Magnetic resonance imaging findings suggestive of ECD. Before starting Vemurafenib, MRI of the brain (**a**, **b**; fluid attenuated inversion recovery: FLAIR sequences) showed cerebellar atrophy and hyperintensity of the dentate nuclei (DN). T2 hyperintense inflammatory lesions (**c**, **d**; arrows) of the DN seen at treatment initiation (**c**) and after 6 months of BRAF inhibition (**d**). Susceptibility weighted imaging (**e**, **f**) demonstrated paramagnetic compound accumulation in the DN (**e**, arrows), caudate nuclei, and globi pallidi (**f**, arrow). Spectroscopy MRI (**g**, **h**) showed a reduction of N-acetyl aspartate (NAA) in the DN (**g**: red box in axial FLAIR MRI was the region of measurements reported in (**h**))

**Fig. 3:**
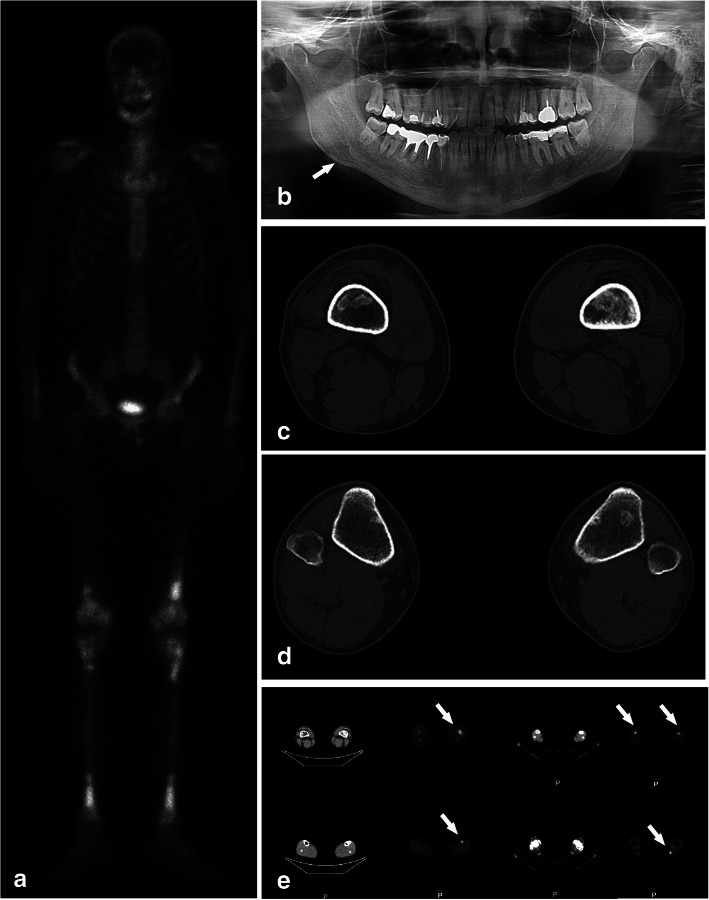
Bone scans findings suggestive of ECD. ^99^Tc-MDP bone scintigraphy (**a**) demonstrating multiple areas of abnormally increased radiotracer uptake including the distal femurs and symmetric uptake in the tibiae, with additional areas of weak uptake in the ulnae, radii, right mandible, left maxilla, thoracic vertebrae. Orthopantomogram (**b**; panorex) demonstrating lucency (arrow) in the right mandible. CT scans of distal portions of the thighs (**c**) and legs (**d**) showing medullary areas of osteosclerosis without cortical erosions in the femurs and tibiae. Similarly, PET (**e**) showing increased FDG uptake in multiple skeletal locations. As in the case of the bone scan, the symmetric uptake in the lower extremities (arrows) is strongly suggestive of ECD

Extensive laboratory workup including complete blood count, complete metabolic panel, hormonal profile, inflammation markers, immunoglobulins, anti-Yo antibody, coeliac disease serology, interferon alpha, angiotensin-converting enzyme, Quantiferon, hepatitis B virus, hepatitis C virus, human immunodeficiency virus, and syphilis testing was unremarkable, except for persistent monocytosis. Low levels of both testosterone and circulating T regulatory lymphocytes were found, and high levels of vascular endothelial growth factor (× 2 upper limit of normal-ULN) and anti-thyroid peroxidase (× 2 ULN), in the presence of anti-nucleus Antibodies (Abs; titer: 1:640) with positive anti-dense fine speckled 70 Abs. To rule out a possible diagnosis of cerebrotendineous xanthomatosis or Gaucher’s disease, plasma cholestanol and chitotriosidase levels were measured, resulting normal and slightly increased (1.8x), respectively. Genetic analysis of spino-cerebellar ataxia *(SCA)1*, *SCA2*, *SCA3*, *SCA6*, *SCA7* and *SCA36* genes did not reveal any mutations, nor was the Friedreich’s ataxia *(FRDA)* trinucleotide repeat disorder identified. MRI of the brain revealed atrophy of the midbrain, cerebellum, superior and middle cerebellar peduncles (Fig. 2a, b). Hyperintense signals were observed in T2 and fluid-attenuated inversion recovery (FLAIR) sequences within the pons, cerebellar white matter, dentate nuclei (DN), and globi pallidi in the absence of any contrast enhancement (Fig. 2c, d, g). Both the retro-orbital space and the pituitary gland appeared normal. Susceptibility weighted imaging (Fig. 2e, f) demonstrated paramagnetic compound accumulation in the DN, caudate nuclei, and globi pallidi. Spectroscopy MRI showed a reduction of N-acetyl aspartate in the DN and globi pallidi (Fig. 2g, h). Whole-body bone scintigraphy with ^99^technetium-methylene diphosphonate (^99^Tc-MDP) showed intense, abnormal radiotracer activity in the distal femurs and whole tibiae bilaterally, with additional areas of weak uptake in the ulnae, radii, right mandible, and left maxilla (Fig. 3a, b). This pattern of skeletal colonization was further confirmed by fluorodeoxyglucose-positron emission tomography (FDG-PET) imaging (Fig. 3e), and high resolution computed tomography (CT) of the femurs and tibiae consistently showed the presence of granulomatous lesions characterized by central demineralization and peripheral osteosclerosis (Fig. 3c, d). A brain ^18^F-Florbetaben PET was carried out to detect the presence of β-amyloid, but was unrevealing, as were CT scan of the thorax and abdomen and MRI of the heart.

The presence of xanthelasma, the biopsy findings, cerebellar degeneration and skeletal findings raised the diagnostic suspicion of ECD. Genetic testing of the formalin-fixed paraffin-embedded skin lesion identified the V600E, GTC > GAG mutation of the *BRAF* gene, further corroborating the diagnostic suspicion. To rule out a concomitant diagnosis of Rosai-Dorfman-Destombes disease [9], a sternal fine-needle biopsy of bone marrow was performed and demonstrated the absence of emperipolesis. Immunostaining for S100 was negative, whereas a Kirsten rat sarcoma (*KRAS*) mutation was found in 1% of nucleated marrow blood cells.

Given the extensive CNS involvement, the patient was started on the BRAF inhibitor Vemurafenib at 960 mg bid. After 15 months, the patient is continuing on the same treatment. Multiple dose adjustments have been necessary in this timeframe as a result of the poor individual drug tolerance. Asthenia, weight loss, and palmoplantar fibromatosis have been the main adverse events. Clinically, the patient has benefited from the BRAF blockade, with disappearance of the xanthelasma and a progressive improvement of the cerebellar symptoms. Nonetheless, the CNS and skeletal findings have remained substantially unchanged, as demonstrated by follow-up imaging. The possibility of combining an anti-MAP2K1 agent with Vemurafenib was recently refused by the patient.

## Discussion and conclusion

The neurological presentation of this neuro-ECD case bears some similarities to, but also differences from previously reported cases of ECD. Indeed, while pyramidal and extrapyramidal symptoms are frequently reported in patients with ECD [6, 10], the occurrence of myasthenia-like symptoms with evening fatigability has never previously been described in this patient population, to the best of our knowledge. The perception of difficulty in sustaining voluntary activities is a typical symptom of cancers [11], also with *BRAF*^*V600E*^ mutation [12], of different neurological diseases [13], comprising inherited cerebellar ataxia [14] and ECD [4]. Fatigue could be associated with performance fatigability of repetitive movements [13], which instead is typical of myasthenia gravis and muscle disorders. The fatigability has been rarely related to cerebellar dysfunction [15, 16], but could be caused in this patients by altered connections between cerebellum and prefrontal cortex [17] or bilateral glodi pallidi dysconnection [18]. In patients with myasthenia-like symptoms who tested negatively for myasthenia gravis, neurodegenerative diseases [19] comprising inherited and autoimmune cerebellar ataxia [14, 20], and disorders of the hypothalamus [21], a diagnosis of ECD should be taken into consideration.

Both cognitive impairment and bilateral neurosensorial hypoacusia were diagnosed at presentation in our patient. The presence of cognitive impairment has been reported in one third of neuro-ECD patients [2], but the pathogenesis is still poorly understood. In a recent analysis of patient-reported outcomes of 50 prospectively enrolled ECD patients, attention and memory appeared impaired in 52% of the cohort [22]. A retrospective volumetric neuroimaging study of 11 ECD patients without recognizable supratentorial CNS lesions demonstrated a substantial reduction of cerebral gray matter volumes in ECD patients as compared to matched controls [23]. These neuro-ECD aspects remain unclear and are the subject of a prospective study (NCT03127709), but a possible diagnosis of ECD should be considered in the presence of progressive cognitive deterioration occurring in adult patients with no risk factors for early-onset dementia. Progressive cerebellar cognitive affective syndrome has been associated with DN and dentato-thalamic pathway degeneration in FRDA disease [24]. Hearing loss has been reported in up to 8% of ECD patients with skull base lesions [25]. However, no tumor-like masses were identified in our patient, in whom only cerebellar atrophy and T2 hyperintense inflammatory lesions of the DN were observed. Since these findings are similar to the inflammatory neurodegeneration seen in typical Langerhans cell histiocytosis, shared neurodegeneration mechanisms might be envisaged.

The absence of typical features of ECD such as cardiovascular and retroperitoneal involvement [3, 5], together with the rarity of the disease, might have contributed to the diagnostic delay in our patient. At present, there is no definite evidence that a timely initiation of treatment might improve the survival outcomes of ECD patients. However, it is conceivable that an increased awareness of the disease and its protean manifestations might increase the diagnostic capability of physicians, thus reducing the diagnostic lag in order to alleviate the psychological burden of patients suffering from an ill-characterized disease.

The treatment of ECD has evolved dramatically in recent years. However, multiple questions remain unanswered. What is the best sequence of treatment? What is the optimal duration of therapy? Is there any biomarker capable of guiding personalized treatment decisions? What are the long-term adverse events associated with BRAF or MEK inhibitors in ECD patients? Future research is needed to address these issues.

## Data Availability

Data sharing is not applicable to this case report due to the risk of breaking individual privacy.

## Abbreviations

^99^Tc MDP: ^99^ Technetium methylene diphosphonate
Abs: Antibodies
BRAF: B-type of Rapidly Accelerated Fibrosarcoma protein kinase
CNS: Central nervous system
CT: Computed tomography
DN: Dentate nuclei
ECD: Erdheim-Chester disease
FDG-PET: Fluorodeoxyglucose-positron emission tomography
FLAIR: Fluid-attenuated inversion recovery
FRDA: Friedreich’s ataxia
H&E: hematoxylin and eosin
KRAS: Kirsten rat sarcoma
MAP2K1: Mitogen-activated protein kinase kinase 1
MRI: Magnetic resonance imaging
PET: Positron emission tomography
SCA: Spino-cerebellar ataxia
ULN: Upper limit of normal
